# Effectiveness of COVID-19 bivalent vaccination against SARS-CoV-2 infection among residents of US nursing homes, November 2022 – March 2023

**DOI:** 10.1016/j.vaccine.2024.07.013

**Published:** 2024-07-16

**Authors:** Kelly Hatfield, Ryan Wiegand, Sujan Reddy, Arshiya Patel, James Baggs, Thomas Franceschini, Amber Gensheimer, Ruth Link-Gelles, John Jernigan, Megan Wallace

**Affiliations:** a Division of Healthcare Quality Promotion, National Center for Emerging and Zoonotic Infectious Diseases, Atlanta, GA, United States; b Coronavirus and Other Respiratory Viruses Division, National Center for Immunizations and Respiratory Diseases, Centers for Disease Control and Prevention, Atlanta, GA, United States; c Signature Healthcare, Louisville, KY, United States; d Chenega Enterprise, Systems, and Solutions, Anchorage, AK, United States

**Keywords:** COVID-19, SARS-CoV-2 infection, Nursing home, Vaccine effectiveness

## Abstract

**Background:**

Residents of nursing homes remain an epidemiologically important population for COVID-19 prevention efforts, including vaccination. We aim to understand effectiveness of bivalent vaccination for preventing SARS-CoV-2 infections in this population.

**Methods:**

We used a retrospective cohort of nursing home residents from November 1, 2022, through March 31, 2023, to identify new SARS-CoV-2 infections. A Cox proportional hazards model was used to estimate hazard ratios comparing residents with a bivalent vaccination compared with residents not up to date with vaccination recommendations. Vaccine effectiveness was estimated as (1 – Hazard Ratio) * 100.

**Results:**

Among 6,916 residents residing in 76 nursing homes included in our cohort, 3,211 (46%) received a bivalent vaccine 7 or more days prior to censoring. Adjusted vaccine effectiveness against laboratory confirmed SARS-CoV-2 infection comparing receipt of a bivalent vaccine versus not up to date vaccine status was 29% (95% Confidence interval 18% to 39%). Vaccine effectiveness for receipt of a bivalent vaccine against residents who were unvaccinated or vaccinated more than a year prior was 32% (95% CI: 20% to 42%,) and was 25% compared with residents who were vaccinated with a monovalent vaccine in the past 61–365 days (95% CI:10% to 37%).

**Conclusions:**

Bivalent COVID-19 vaccines provided additional protection against SARS-CoV-2 infections in nursing home residents during our study time-period, compared to both no vaccination or vaccination more than a year ago and monovalent vaccination 60 – 365 days prior. Ensuring nursing home residents stay up to date with vaccine recommendations remains a critical tool for COVID-19 prevention efforts.

## 1. Introduction

Residents of nursing homes have experienced disproportionate morbidity and mortality due to COVID-19 and substantial disruption to daily living [1–3]. Continued prevention efforts are important for nursing home residents because of their additional risk for infection and subsequent poor outcomes due to their congregate living status, advanced age, and often decreased health status with comorbid conditions. However, because of some of these same factors, the effectiveness of COVID-19 vaccines may be different in nursing home residents compared with community-dwelling adults. Vaccine effectiveness (VE) of monovalent mRNA vaccines against SARS-CoV-2 infection has been shown to wane more quickly and to be lower against more recently emerged Omicron variants of SARS-CoV-2 compared with previous lineages including Delta [4,5]. Due to higher COVID-19-associated morbidity and mortality in nursing home residents, understanding the vaccine effectiveness in this population is critical for vaccine policy recommendations and targeted prevention efforts. Previous research in this population has shown the relative effectiveness of a monovalent COVID-19 mRNA booster dose against infection compared with primary series vaccination alone ranged from approximately 35 to 60% against SARS-CoV-2 infection with Delta and Omicron variants [6,7].

In August 2022, the FDA authorized Pfizer-BioNTech and Moderna bivalent (ancestral and Omicron BA.4/BA.5 variant) COVID-19 vaccines for use as booster doses, and in September 2022, the CDC recommended bivalent booster doses for all adults ≥ 2 months after last primary series or monovalent booster dose [8]. Early estimates among immunocompetent adults suggested that bivalent doses provided additional protection against symptomatic infection [9]. There are limited data on the effectiveness of bivalent vaccination among nursing home residents. An analysis of facility level data from the National Healthcare Safety Network found VE against infection of 31% [10]. The objective of this analysis is to estimate effectiveness of bivalent vaccination against infection using resident-level data from nursing homes.

## 2. Methods

Data from 76 nursing homes in eight states operating under the tradename Signature Healthcare, predominantly from the Southeast region, were compiled to ascertain resident census, vaccination, and SARS-CoV-2 testing information. Data were collected for any resident who had been present in a participating nursing home from November 1, 2022, through March 31, 2023, and included: dates present at a facility, demographic characteristics, COVID-19 vaccination dates, vaccine product, and SARS-CoV-2 testing information (test date, type, and result). International Classification of Diseases 10th revision, clinical modification (ICD-10-CM) codes and corresponding onset dates were extracted from nursing home electronic medical records for each resident in the cohort.

### 2.1. Cohort description

We created a retrospective cohort of residents who were living in a participating nursing home for at least one day in our study period. We selected November 1, 2022, to begin our assessment as it marked the date that 75% of participating nursing homes had at least 3 residents receiving a bivalent dose in their facility. Residents with a positive SARS-CoV-2 test (antigen test and/or reverse transcription polymerase chain reaction (RT-PCR) or a COVID-19 ICD-10-CM diagnosis code (U07.1)) on the day of or within the 30 days preceding our study period were excluded. Due to potential differences in documentation of comorbidities and vaccination status, residents who spent less than 30 days in the nursing home during our entire study period were excluded.

Demographics including age, gender, payer for nursing home stay, underlying medical conditions, testing intensity, and history of COVID-19 were extracted from electronic health record data. The presence of one or more underlying medical conditions that are risk factors for severe COVID-19 were defined based on ICD-10-CM diagnosis codes present in the electronic health record and included cancer, chronic kidney disease, chronic obstructive pulmonary disease, heart conditions, immunocompromised states, chemotherapy, radiation, obesity, sickle cell disease, smoking, and diabetes [11]. Using a variable describing the payer for each day of the nursing home stay, we identified residents covered by Medicaid and those receiving hospice care. Average testing intensity for each resident was calculated as the number of tests throughout the study period per 30 days present. History of SARS-CoV-2 infection was defined as a positive SARS-CoV-2 antigen or RT-PCR test or a COVID-19 ICD-10-CM diagnosis code (U07.1) in the electronic health record using the date of test or date of diagnosis recorded in the electronic health record from 31 – 180 days preceding the study start date.

### 2.2. Vaccination status

Beginning in September 2022, participating nursing homes implemented practices where nursing home personnel validated current and historical vaccination status for all residents through manual review of the electronic health record (including free-text notes fields), review of CDC COVID-19 vaccine status cards, and search of jurisdictional-based immunization information systems (IIS). Simultaneously, the nursing homes implemented practices where nursing home personnel also established enhanced protocols for vaccination history review at admission that were ongoing throughout our study period. In mid-January 2023 (middle of our study period), we conducted a validation of a subset of nursing home residents in one state who resided in the nursing home between October 1, 2022, and January 15, 2023. We validated vaccination records compared with the IIS for 4,864 residents. At that time, only 94 (<2%) had additional bivalent booster data recorded in the IIS that occurred during their time present in a Signature nursing home and did not match what was reported in the electronic health record, providing sufficient confidence in quality of the electronic health record vaccination data.

### 2.3. Resident follow-up

Resident-time follow up started on November 1, 2022, or the first date present in a participating nursing home in the study follow up period and continued through March 31, 2023, until a resident experienced a positive SARS-CoV-2 test, or the resident left the nursing home (censoring event). Residents were not included in the analysis on days they were not present in a participating facility but could reenter the cohort on readmission into the same facility. Vaccination status was considered time dependent; thus, all resident-days were categorized by vaccination status, and residents could have varied vaccination status throughout the study period. Resident-day vaccination status was assigned as not up to date if the resident was unvaccinated (i.e., no documented date of receipt for any COVID-19 vaccine), vaccinated with a monovalent vaccine more than 365 days prior, or vaccinated with a monovalent vaccine 61 – 365 days prior. Other resident-day vaccination statuses included receipt of a monovalent vaccine in the prior 0 – 60 days, receipt of a bivalent vaccine in the past 0 – 6 days, and receipt of a bivalent vaccine 7 or more days prior. Resident-time where a resident had received a monovalent vaccine in the past 0 – 60 days (i.e., not eligible for a bivalent vaccine) or received a bivalent vaccine in the past 0 – 6 days (i.e., before dose protection was fully conferred) was excluded from models. Among residents with a bivalent vaccine, we described the time since dose receipt at the study start and end dates.

### 2.4. SARS-CoV-2 testing

SARS-CoV-2 testing was conducted according to nursing home protocols and was similar to CDC’s recommended testing strategies in nursing homes [12]. SARS-CoV-2 testing was conducted for residents with new-onset COVID-19-like symptoms or after exposure to a COVID-19 positive person (testing occurred at 24 h, day 3, and day 5 after contact). Further, when community transmission rates were high, new resident admissions were tested at admission, on day 3, and on day 5 of their stay. The nursing home policy was not to test residents who were previously positive in the past 30 days. Information for all SARS-Cov-2 testing performed by the nursing home, including date, type of test (i.e., antigen point-of-care or RT-PCR), and result for all tests administered throughout the study period, was ascertained.

### 2.5. Statistical analyses

We compared descriptive statistics for resident’s age, gender, number of underlying medical conditions, length of stay, number of SARS-CoV-2 tests, and history of COVID-19 among those with and without a positive test using Chi-square tests for categorical variables and two sample t-tests for continuous variables. Incidence rates of SARS-CoV-2 infections per 10,000 resident days were calculated for the entire study period and using a 30-day moving average. We used multivariable Cox proportional hazards models using a log-normal frailty distribution accounting for clustering within a nursing home and with a time-dependent vaccination status to estimate hazard ratios (HRs) for time to first positive SARS-CoV-2 test in our study period. Hazard ratios compared rates of SARS-CoV-2 infection among residents receiving a bivalent dose to rates among residents with other vaccination statuses after accounting for clustering within a nursing home and adjusting for resident’s age, gender, number of underlying medical conditions, and history of COVID-19 as fixed effects. We calculated VE against the outcome of laboratory confirmed SARS-CoV-2 infection (i.e., a positive test for SARS-CoV-2) as (1- HR)*100%. By using the hazard ratios to calculate VE, we assume that the effectiveness is constant over time. We calculated VE using two models with varying exposure and reference groups. Model one evaluated any receipt of bivalent dose compared to a population not up to date. A sub-analysis for model one excluded patients receiving hospice care. To describe the effect of vaccination relative to time since previous vaccination, model two assessed any receipt of a bivalent dose to two reference populations. First, we calculated the VE of receipt of a bivalent dose versus no vaccine in the prior year (i.e., unvaccinated and vaccinated more than one year prior). Second, we calculated the VE of receipt of a bivalent dose versus a monovalent vaccine 2 months to 1 year prior.

Analyses were conducted using SAS version 9.4 (SAS Institute) using the 5% level of significance. This activity was determined by CDC to be non-research (such that it does not require patient consent) per 45C.F.R. part 46.102(l)(2) and was conducted consistent with applicable federal law and CDC policy.

## 3. Results

**Fig. 1.**
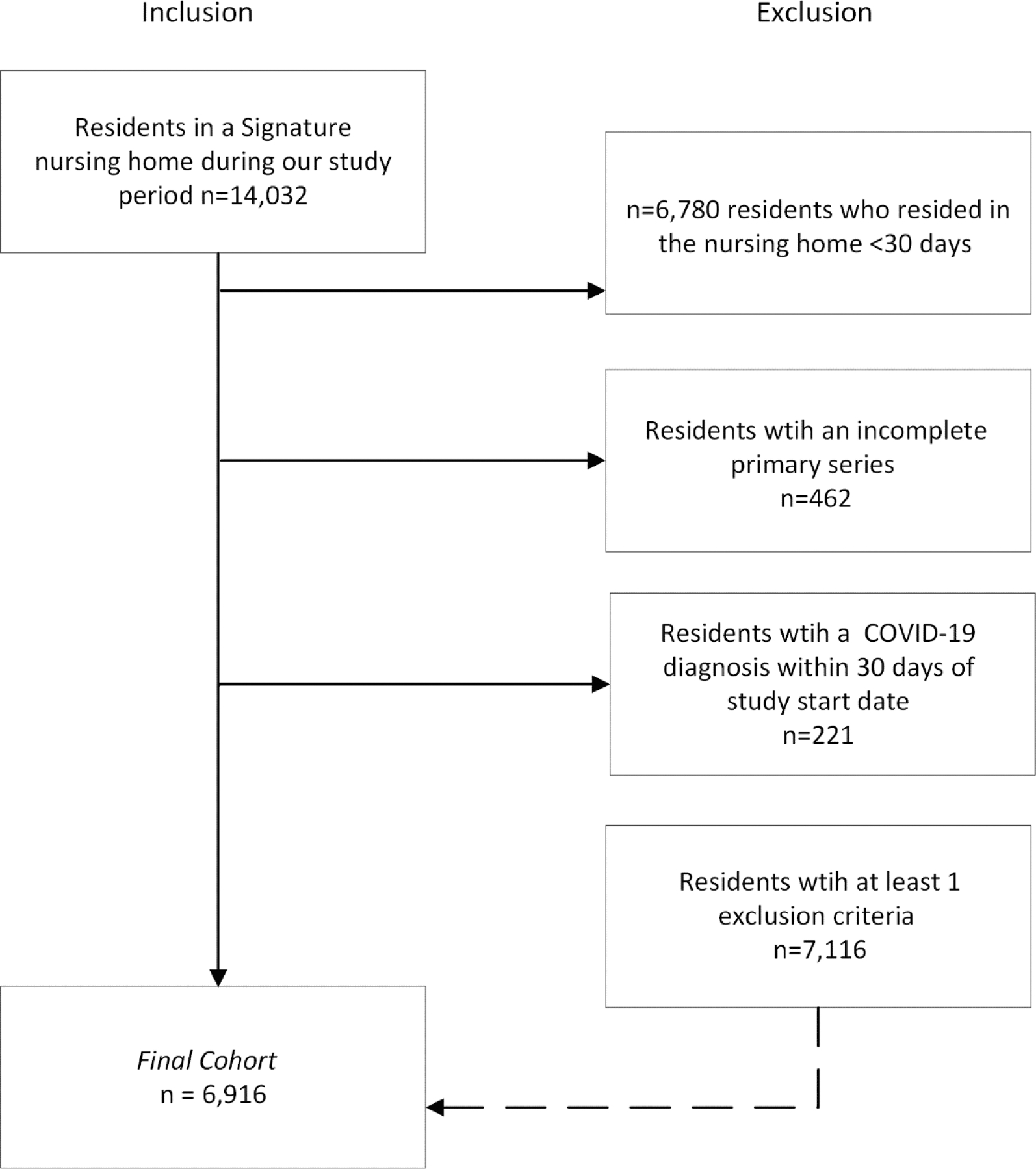
Flow diagram of residents of Signature nursing homes from November 1, 2022 – March 31, 2023.

Data for 14,032 residents who were present for at least one day in 76 participating nursing homes were reviewed to determined eligibility for inclusion. Of these, 6,916 (49%) met inclusion criteria for our cohort (Fig. 1). Nursing homes were located in Kentucky (40 homes), Tennessee (21), Indiana (4), Ohio (4), North Carolina (3), Georgia (2), Alabama (1), and Virginia (1); a median average daily census of 90 residents per nursing home were included throughout the study period (interquartile range [IQR]: 70–113). More than half of the residents in the cohort were female (n = 4,346, 63%); 4,268 (62%) had diagnosis codes representing one or more of the underlying medical conditions assessed, and 816 (13%) residents had a prior COVID-19 diagnosis or a positive SARS-CoV-2 test in the 31 – 180 days before our study period. The majority (n = 5,104 74%) of residents were long stay status (i.e., 90 or more days present from January 1, 2022-March 31, 2023). A minority (n = 393, 6%) of residents had documented hospice status during the study period, while nearly three in four (5,050, 73%) had Medicaid pay for at least one day during the study period. Residents contributed a median of 135 resident-days (IQR: 61, 152) to the analysis, and residents had an average of 3.7 tests throughout the study period.

**Fig. 2.**
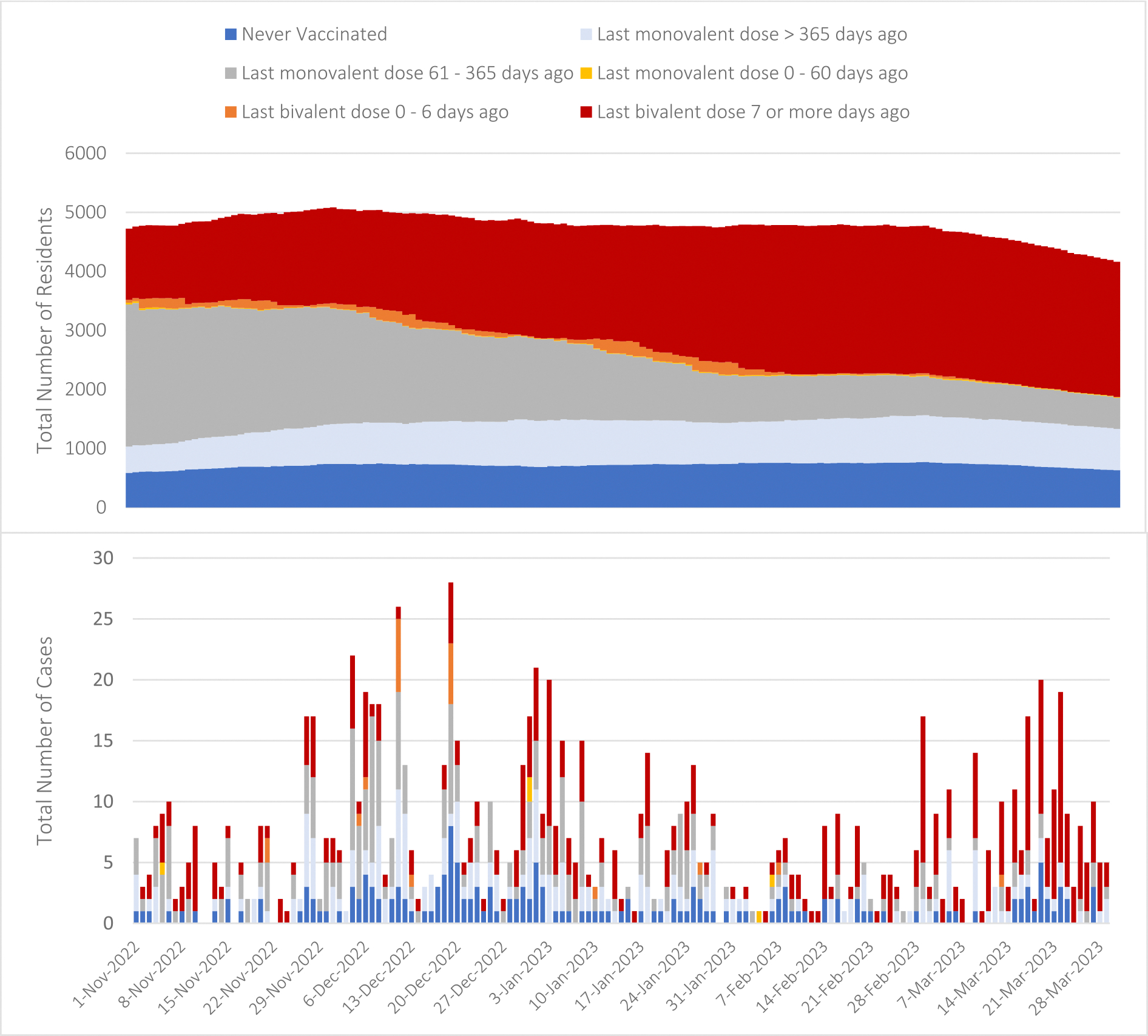
Vaccination status for each resident included in the cohort (top panel) and each incident SARS-CoV-2 infection (bottom panel), by day, November 1, 2022, through March 31, 2023.

Vaccination status for all residents present each day are shown in Fig. 2a; 26% of residents had received a bivalent vaccine more than 7 days ago at the study start (November 1, 2022), compared with 55% at the study end (March 31, 2023). At censoring (i.e., the end of the study period for each resident), 3,255 (47%) residents had received a bivalent vaccine; among those, 44 had received the vaccine 0–6 days prior (Table 1). An additional 3,617 (53%) residents were not up to date with vaccine recommendations at censoring: 1,235 (18%) were never vaccinated, 1,370 (20%) had received a monovalent vaccine over a year prior, and 1,032 (15%) had received a monovalent vaccine 61–365 days prior and were therefore eligible for (but had not received) a bivalent vaccine dose. There were 24 (<1%) residents who had received a monovalent vaccine in the past 0–60 days (and thus were not eligible for a bivalent vaccine) at censoring.

Among 3,255 residents who received a bivalent vaccine prior to censoring, the date of receipt spanned from vaccine authorization in September 2022 throughout the study period to March 2023. At the beginning of the study period (November 1, 2022), 1,437 (44%) of residents who received a bivalent vaccine prior to censoring were within 60 days of receiving their bivalent vaccine and 1,818 (56%) had not yet received a bivalent vaccine. By the end of the study period (March 31, 2023) that distribution had shifted; 211 (6%) of the residents that received a bivalent vaccine prior to censoring were within 60 days of receiving their dose, 1,102 (34%) were within 61 – 120 days, 1,328 (41%) were within 121 – 180 days, and 614 (19%) were more than 180 days since their bivalent vaccine.

Overall, 1,009 (15%) residents had a positive test for SARS-CoV-2 during the study period (Fig. 2b). Another 1,748 (25%) residents were censored due to leaving the participating nursing home prior to the end of the study period, and 4,159 (60%) remained in the nursing home at the end of the study period (March 31, 2023). Compared to residents without a positive test in our study period, those with a positive test were less likely to have hospice or Medicaid payers during the study period (Hospice: 3% for positive test versus 6% without positive test, p < 0.001, Medicaid: 67% versus 74%, p < 0.001) and more likely to have no prior history of COVID-19 (67% for residents with a positive test versus 53% without, p < 0.001, Table 1). Residents with a positive test were tested more often than residents without a positive test; residents with a positive test had an average of 4.5 tests throughout the study period (95% CI: 4.2, 4.8) compared to 3.5 for residents without a positive test (95% CI: 3.4, 3.6, p < 0.001, Table 1). Vaccination status at censoring also varied significantly by test status (Table 1, p < 0.001).

**Fig. 3.**
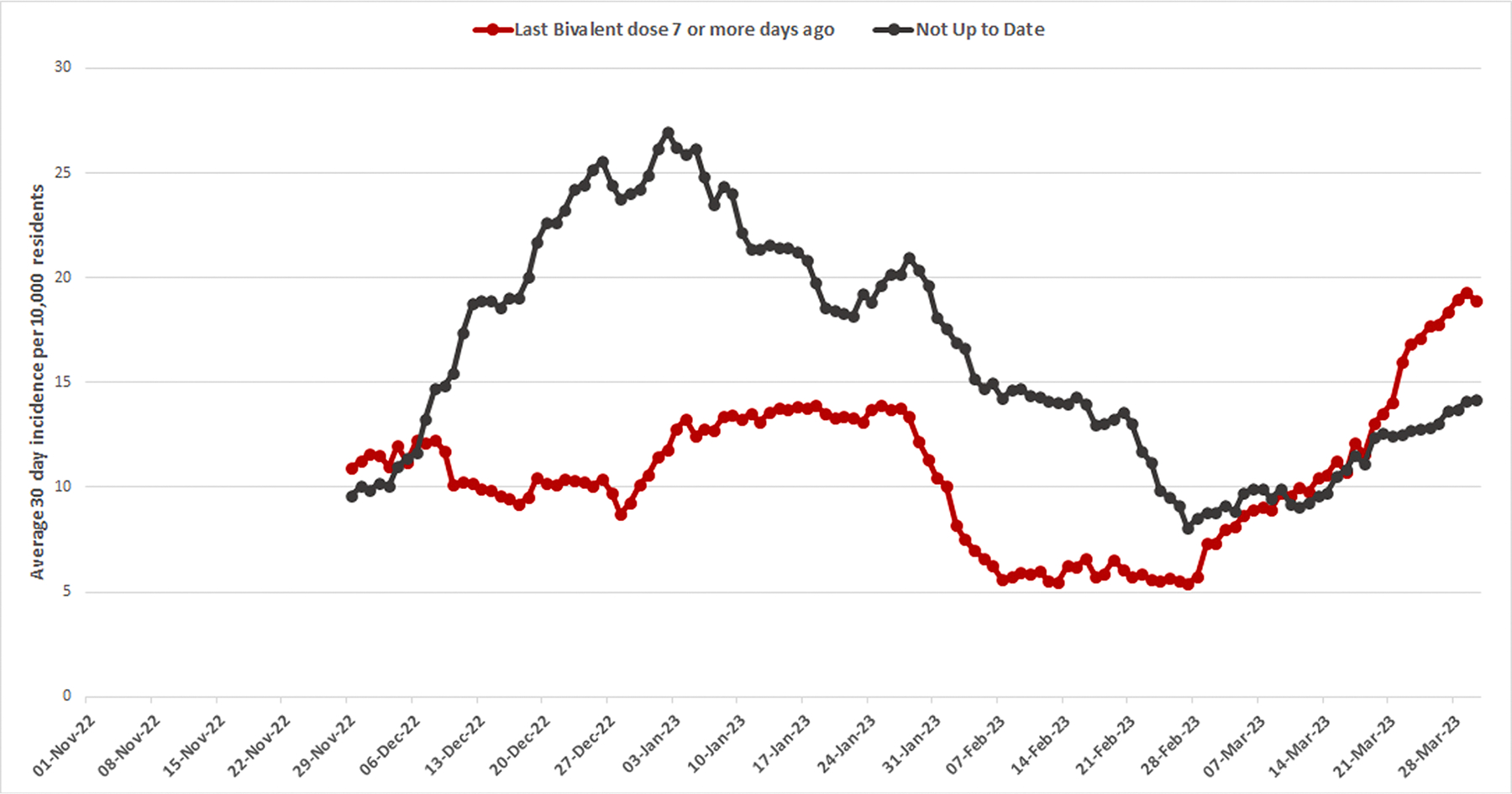
30-day moving average incidence of SARS-CoV-2 infections per 10,000 residents stratified by vaccination status. *Not up to date includes residents who were never vaccinated (i.e., no documented date of receipt for any COVID-19 vaccine), vaccinated with a monovalent vaccine more than 365 days prior, or vaccinated with a monovalent vaccine 61 – 365 days prior.

Among the vaccination status groups included in our models, SARS-CoV-2 infection rates were highest among residents who had received their last vaccine dose more than 1 year prior (18.9 per 10,000 resident-days) and lowest for residents who had received a bivalent vaccine more than 7 days prior (11.6 per 10,000 resident days, Table 2). Moving 30-day average incidence shows lower rates among residents who had received a bivalent vaccine more than 7 days prior throughout most of the study period, however SARS-CoV-2 infection rates increase higher compared to residents who are not up to date beginning in March 2023 (Fig. 3).

Adjusted models identified significant associations with history of COVID-19, gender, and comorbid conditions with the hazard of SARS-CoV-2 infection in our study (Table 3). Adjusted VE from model one assessing receipt of a bivalent vaccine versus not up to date vaccine status was 29% (95% CI: 18% to 39%, Table 4). We found similar estimates when we excluded residents who were receiving hospice care from the model (VE: 28%, 95% CI: 17% to 38%). From model two, we demonstrated significant VE for receipt of a bivalent vaccine compared to no vaccine or vaccination more than one year prior (VE: 32%, 95% CI: 20% to 42%,) and compared to receipt of a monovalent vaccine in the past 61–365 days (VE: 25%, 95% CI: 10% to 37%) (Table 3). Model two did not show a statistically significant protection of monovalent vaccination in the past 61 – 365 days versus unvaccinated or vaccinated more than a year prior (VE: 10%, 95% CI: −8% to 24%) in our study period.

## 4. Discussion

Our findings demonstrate that bivalent vaccination provided additional protection against SARS-CoV-2 infection in a large cohort of nursing home residents compared to receiving only monovalent vaccine doses or being unvaccinated. Vaccine effectiveness appeared to be highest (32%) after receipt of a bivalent vaccine compared to residents who were unvaccinated or vaccinated more than a year ago, likely due to lower residual protection from monovalent doses. However, we also identified significant effectiveness (25%) from receipt of a bivalent vaccine compared to residents who had received a monovalent vaccine 60 – 365 days prior. Although we were unable to assess waning vaccine effectiveness of the bivalent vaccine, 60% of residents were over 120 days out from their bivalent dose at the end of the study period, and rates of SARS-CoV-2 infection among residents with bivalent vaccination 7 or more days prior began to match or exceed those of residents who were not up to date later in the study period. Waning may have contributed to a lower overall VE estimate compared to a study that measured effectiveness only in a period closer in time to the receipt of the vaccination. These findings are consistent with a prior analysis demonstrating the effectiveness of bivalent doses against symptomatic infection compared to receipt of monovalent doses only among community dwelling U.S. adults aged 65 years and older and that found that VE started at 38% in the 2 weeks to 1 month after the bivalent dose and waned to 21% by 4–5 months after the bivalent dose [13].

We were not able to assess the effectiveness of bivalent COVID-19 vaccination against severe disease, hospitalization, or death. Measuring outcomes such as severe disease, hospitalization, and death in nursing home settings is often challenging. First, we did not have standardized documentation of clinical data available to ascertain symptom status. Second, we did not have data available to measure hospitalization or mortality rates among nursing home residents. Measuring the risk of hospitalization among nursing home residents requires careful consideration and risk adjustment as previous work has shown hospitalization is often very discretionary with large amounts of over and underutilization [14], and may be dependent on patient preferences, provider attitudes, and financial implications, among other factors [15]. Measuring mortality in the nursing home setting is often difficult due to the inability to follow residents when they leave the nursing home (e.g., mortality after transfer home, or to an acute care facility, or to another care location), which may lead to differential bias or underestimation of mortality rates. Previous experience suggests that COVID-19 vaccination provides even greater and more sustained effectiveness against more severe COVID-19 outcomes such as severe disease, hospitalization, and death, than it does against infection [16–19], suggesting even greater potential value of bivalent vaccine in the nursing home population.

Our findings of non-significant effectiveness of monovalent vaccine against positive tests for SARS-CoV-2 aligns with results in younger, community dwelling adults and earlier vaccine effectiveness estimates in this cohort of nursing homes that demonstrated declining VE from an initial primary series over time and with the emergence of new variants [20]. This is further supported by additional studies which demonstrated the attenuation of protection of monovalent vaccine against symptomatic illness during Omicron predominance [5]. The waning of vaccine effectiveness seen for monovalent doses and the increased protection demonstrated following receipt of a bivalent dose emphasize the importance of bivalent vaccination as an important component of an effective infection control strategy for preventing outbreaks in the nursing home setting. In April 2023, CDC recommended a single, optional additional bivalent dose ≥ 4 months after the most recent bivalent dose for adults aged ≥ 65 years.

This study is subject to several limitations. First, to minimize potential misclassification of exposure for vaccine dose information for residents, we partnered with participating nursing homes to verify vaccination status. However, there is still the potential for vaccine doses to be unreported and resident vaccination status to be misclassified. Second, our statistical power to evaluate VE by time since booster receipt is limited as most residents included in the study cohort received their booster during the same window of time and during our study period. Third, despite efforts to identify prior SARS-CoV-2 infection using both positive tests and COVID-19 diagnosis codes, previous infection may be undocumented leading to misclassification. Fourth, our study was limited to facilities predominantly in the southeastern United States, and therefore, results may not be generalizable outside of that area. Fifth, our data suggest that residents with a positive test were tested slightly more frequently than those without a positive test, potentially indicating some testing bias. However, this testing bias may be appropriate if residents were living in outbreak settings due to the testing procedures by the nursing home and CDC recommended practices (i.e., to test residents more frequently in an outbreak setting). Additionally, our cohort study design accounted for clustering within nursing homes, which should adjust for differences between nursing home levels of transmission and testing practices. Sixth, because of data limitations we estimated vaccine effectiveness against any positive test for SARS-CoV-2. Other studies have suggested increased measures of vaccine effectiveness against more severe outcomes [16–18] that we did not assess.

Over half of our residents who received a bivalent booster prior to censoring received their bivalent booster during our study period, and by the end of our follow up period 76% of our residents who had received a bivalent booster had received it more than 60 days prior. Future assessment of this population should be done to compare effectiveness and assess changes in effectiveness by time since bivalent booster receipt.

This study validates ongoing efforts to ensure nursing home residents stay up to date with vaccination recommendations by demonstrating effectiveness of a bivalent dose at preventing infection. Nursing home administrators and staff should continue to support and encourage receipt of bivalent vaccine in this population.

## Figures and Tables

**Table 1:** Demographics for 6,461 residents included in our cohort stratified by any positive test for SARS-CoV-2 during study period.

	Overall	Positive test for SARS-CoV-2 during study period No. residents (row %)	No positive test for SARS-CoV-2 during study period	

Total	6916 (100%)	1009 (15%)	5907 (85%)	
Person – Time				
Days in study period, Median (Q1 – Q3)	135 (51 to 152)	49 (25 to 85)	147 (61 to 152)	
Censoring Status				
Positive Test (Case)	1009 (15%)	1009 (100%)	0 (0%)	
Discharge/Left Facility	1748 (25%)	0 (0%)	1748 (30%)	
End of Study	4159 (60%)	0 (0%)	4159 (70%)	
Characteristics		No. residents (col %)		p-value
Gender*				0.24
Female	4346 (63%)	611 (61%)	3735 (63%)	
Male	2569 (37%)	398 (39%)	2171 (37%)	
Resident Status, January 1, 2022 – March 31, 2023				0.08
Short Stay (<90 days present)	1812 (26%)	242 (24%)	1570 (27%)	
Long Stay (>= 90 days present)	5104 (74%)	767 (76%)	4337 (73%)	
Age group, yrs				0.15
≤64	1156 (17%)	156 (15%)	1000 (17%)	
65 to 74	1762 (25%)	246 (24%)	1516 (26%)	
75 to 84	2131 (31%)	341 (34%)	1790 (30%)	
≥85	1867 (27%)	266 (26%)	1601 (27%)	
Hospice Status				<0.001
At least one day during study period	393 (6%)	26 (3%)	367 (6%)	
No Hospice during study period	6523 (94%)	983 (97%)	5540 (94%)	
Medicaid Status				<0.001
Yes, at some point during study period	5050 (73%)	677	4373 (74%)	
No Medicaid during study period	1866 (27%)	332	1534 (26%)	
Chronic conditions				0.81
None	2648 (38%)	395 (39%)	2253 (38%)	
1 to 2	2692 (39%)	385 (38%)	2307 (39%)	
≥3	1576	229 (23%)	1347 (23%)	
Number of tests in study period				<0.001
No. Mean, 95% Confidence Interval	3.7 (3.6 to 3.8)	4.5 (4.2 to 4.8)	3.5 (3.4 to 3.6)	
History of COVID-19				<0.001
None	3777 (55%)	676 (67%)	3101 (52%)	
31–180 days prior to study start date	816 (12%)	50 (5%)	766 (13%)	
181–365 days prior to study start date	1072 (16%)	125 (12%)	947 (16%)	
> 365 days ago days prior to study start date	1251 (18%)	158 (16%)	1093 (19%)	
Vaccination status at censoring				<0.001
No monovalent or bivalent doses recorded (i.e., Never vaccinated)	1235 (18%)	169 (17%)	1066 (18%)	
Last monovalent dose received > 365 days prior	1370 (20%)	201 (20%)	1169 (20%)	
Last monovalent dose received 61–365 days prior	1032 (15%)	257 (25%)	775 (13%)	
Last monovalent dose received 0–60 days prior	24 (<1%)	5 (<1%)	19 ( < 1%)	
Bivalent booster received in past 0 – 6 days	44 (1%)	20 (2%)	24 (<1%)	
Bivalent booster received 7 or more days prior	3211 (46%)	357 (35%)	2854 (48%)	

* Gender as reported in the electronic medical record; missing gender variable for one resident.

Comorbid conditions assessed include: cancer, chronic kidney disease, chronic obstructive pulmonary disease, heart conditions, immunocompromised state, chemotherapy, radiation, obesity, sickle cell disease, smoking, and diabetes.

CI: confidence interval. P-values describe results from Chi-square tests for categorical variables and two sample t-tests for continuous variables.

**Table 2:** Resident-days and unadjusted rates of SARS-CoV-2 infections assessed by vaccination status.

Vaccination Status Groups		No. of residents	Median days per Resident (IQR)	Resident-Days	No. of SARS-CoV-2 infections	Rate per 10,000 Resident-days

**Not up to date**	Never vaccinated (i.e., no monovalent or bivalent doses recorded)	1,255	71 (40–149)	108,326	169	15.6
	Last monovalent dose > 365 days prior	1,762	45 (30–86)	106,114	201	18.9
	Last monovalent dose 181–365 days prior	2,891	53 (29–94)	191,347	257	13.4
**Excluded resident-time from Models**	Last monovalent dose 0–60 days prior	61	30 (8–51)	1,971	5	25.4
	Bivalent booster 0 – 6 days prior	1,792	7 (7–7)	12,033	20	16.6
**Up to Date**	Bivalent booster 7 or more days prior	3,211	99 (58–146)	306,865	357	11.6

**Table 3:** Adjusted hazard ratios from multivariable Cox proportional hazards models using a lognormal frailty distribution accounting for clustering within a nursing home and with a time dependent vaccination status.

Covariates	Model 1		Model 2	
	Hazard Ratio	95% Confidence Interval	Hazard Ratio	95% Confidence Interval

History of COVID-19 31–180 days prior to study start date versus no history in that window	0.328	0.243, 0.443	0.330	0.244, 0.446
Gender, Female versus Male	0.807	0.705, 0.923	0.813	0.709, 0.934
Age 18–64 years versus 85+	0.936	0.755, 1.159	0.969	0.779, 1.206
Age 65–74 years versus 85+	1.018	0.846, 1.226	1.044	0.863, 1.263
Age 75–84 versus 85+	1.164	0.984, 1.376	1.192	1.003, 1.415
1 to 2 Comorbid Conditions versus none	0.647	0.556, 0.556	0.651	0.557, 0.557
3 or more Comorbid Conditions versus none	0.717	0.602, 0.855	0.719	0.601, 0.860
Receipt of bivalent dose more than 7 days prior versus not up to date	0.707	0.610, 0.819		
Receipt of Bivalent dose more than 7 days prior versus unvaccinated or receipt of monovalent vaccine > 365 days prior			0.680	0.579, 0.799
Receipt of monovalent vaccine 61––365 days prior versus unvaccinated or receipt of monovalent vaccine > 365 days prior			0.904	0.760, 1.076
Receipt of Bivalent dose more than 7 days prior versus receipt of monovalent vaccine 61–365 days prior			0.752	0.626, 0.904

**Table 4:** Adjusted modeled vaccine effectiveness for three different models from November 1, 2022 - March 31, 2023.

Model	Model Outcome	Reference	Adjusted VE	95% Confidence Interval

**1**	Receipt of Bivalent dose more than 7 days prior	Not Up to Date	29%	18% to 39%
**1** *			28%	17% to 38%
2	Receipt of Bivalent dose more than 7 days prior	Receipt of monovalent vaccine 61–365 days prior	25%	10% to 37%
	Receipt of Bivalent dose more than 7 days prior	Unvaccinated or receipt of monovalent vaccine > 365 days prior	32%	20% to 42%
	Receipt of monovalent vaccine 61–365 days prior	Unvaccinated or receipt of monovalent vaccine > 365 days prior	10%	− 8% to 27%

* Model 1* excludes resident-time while the resident was receiving hospice care.

## Data Availability

The data that has been used is confidential.
